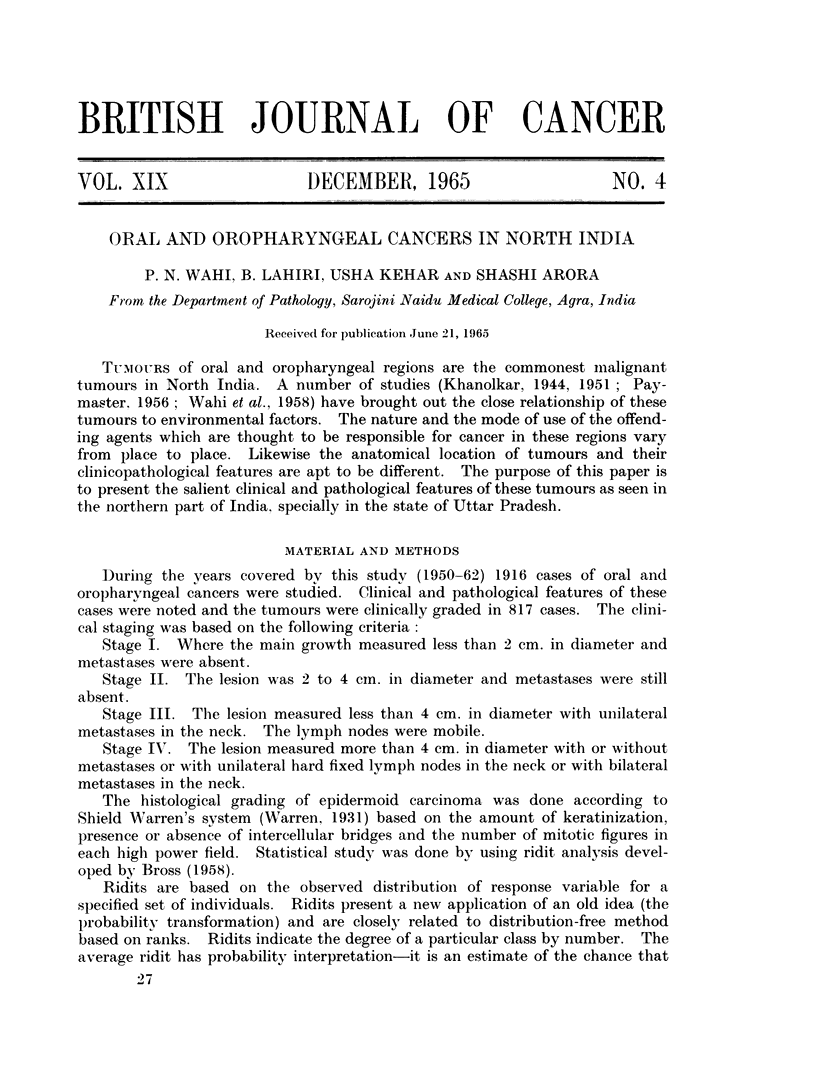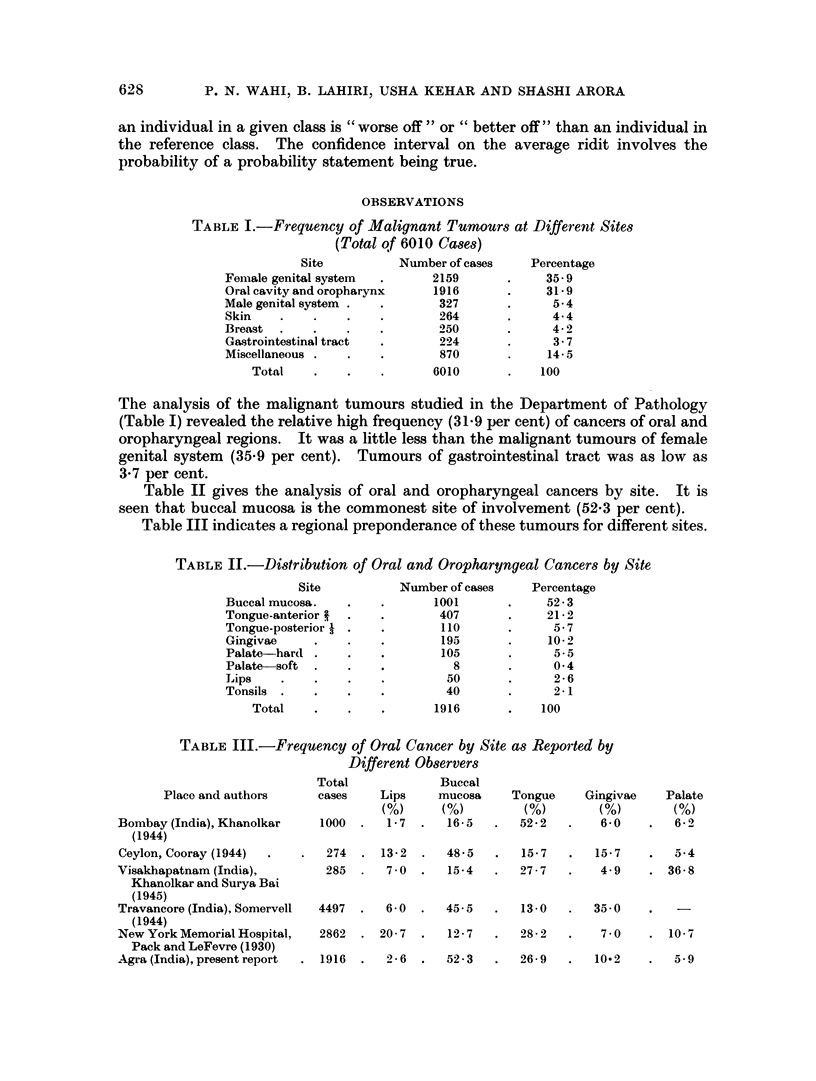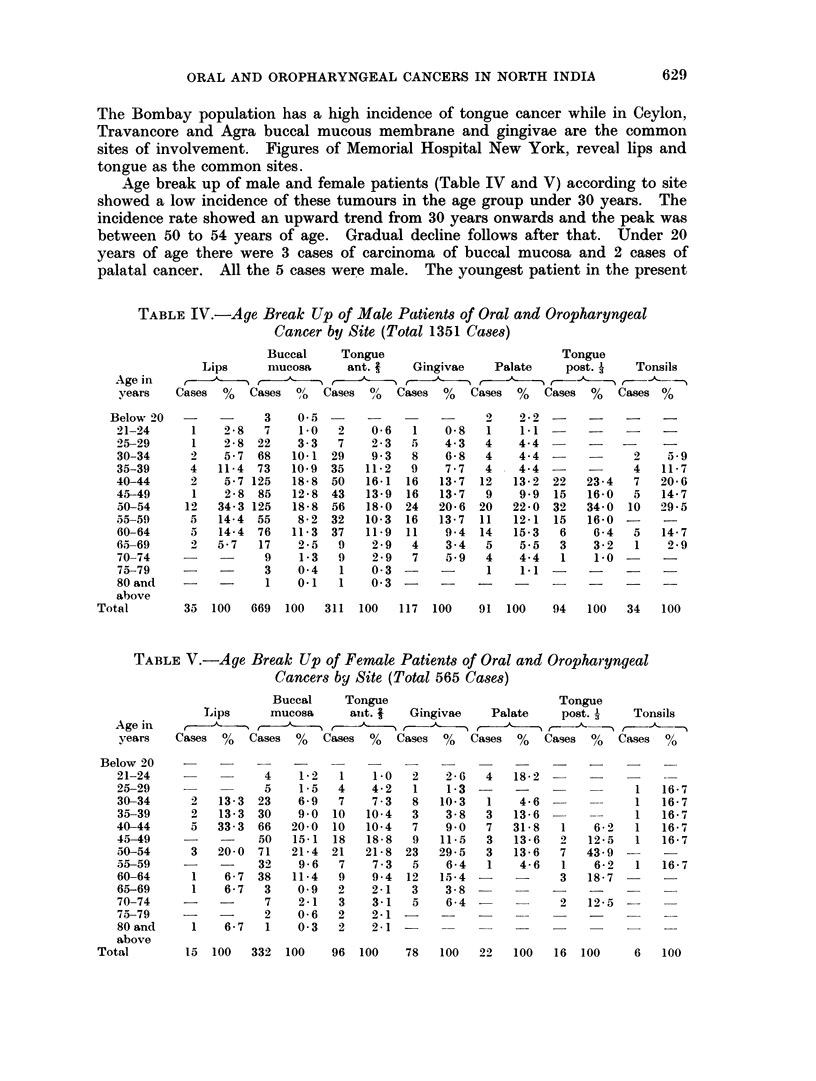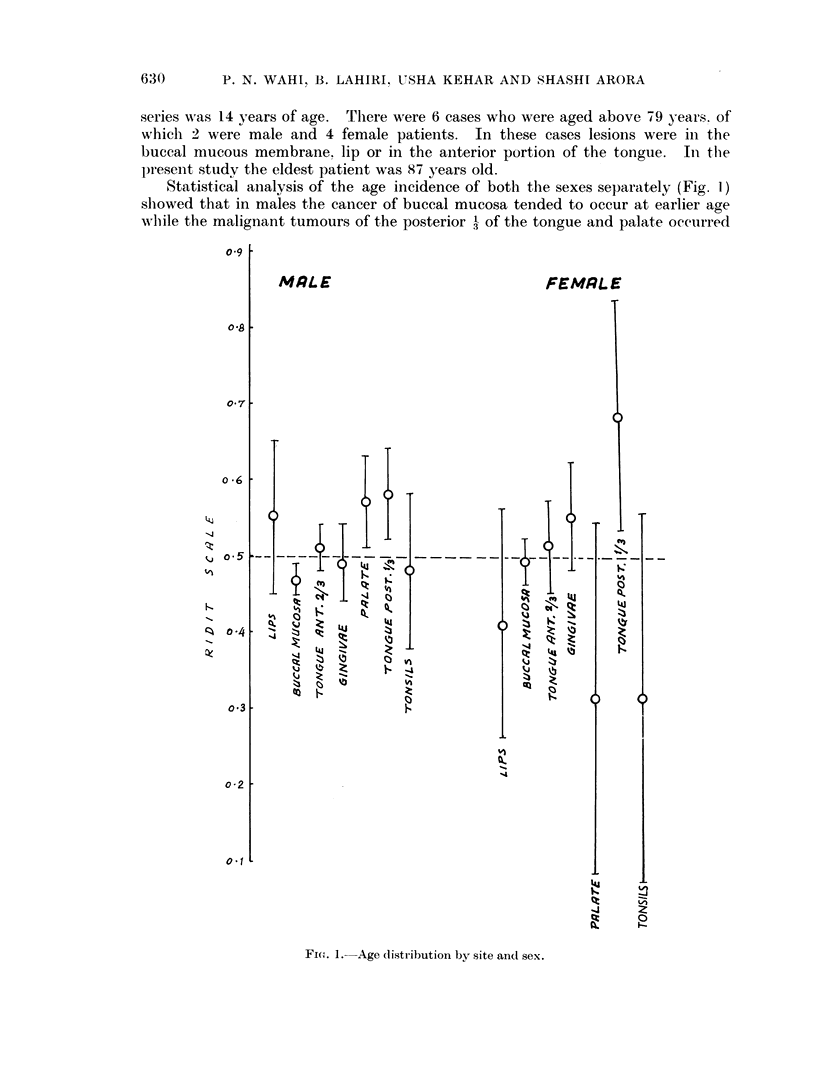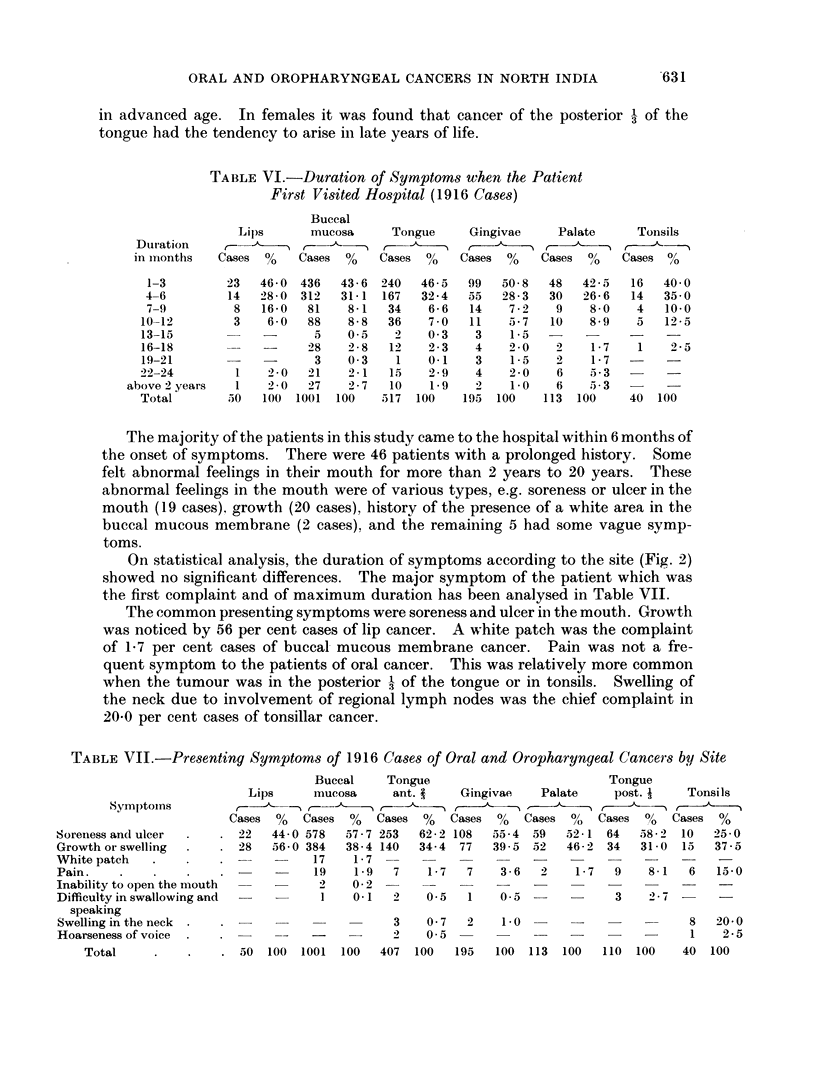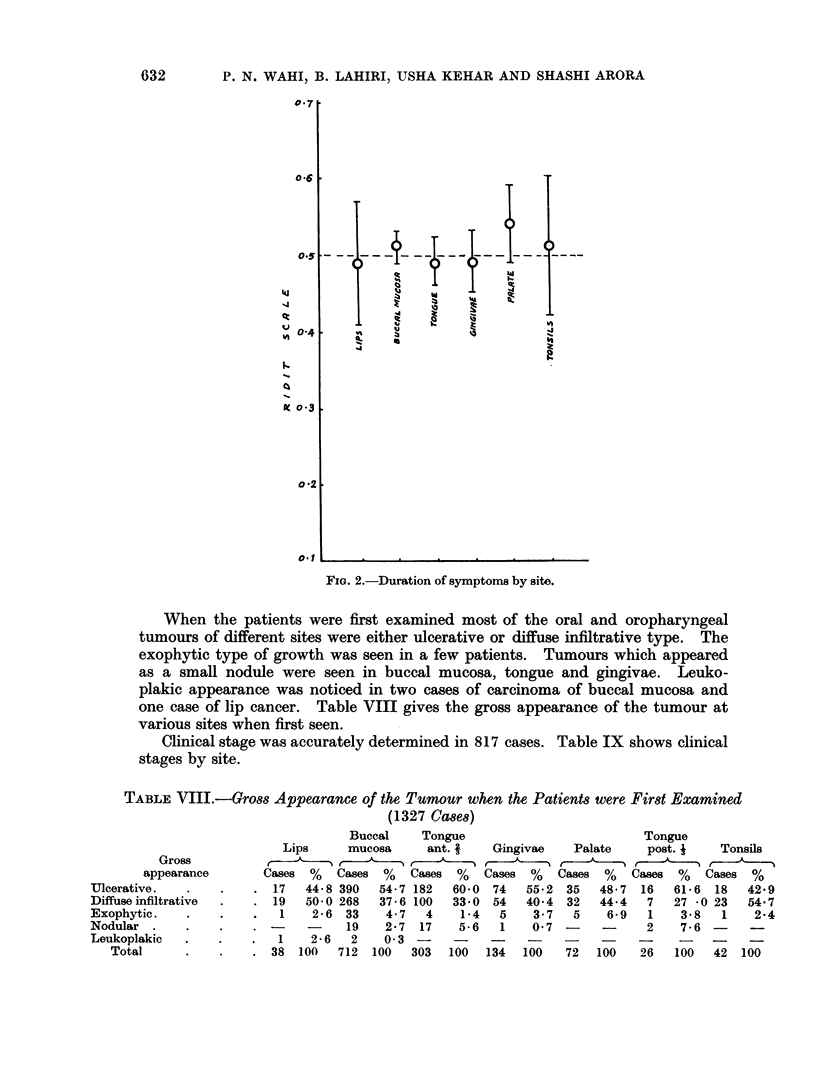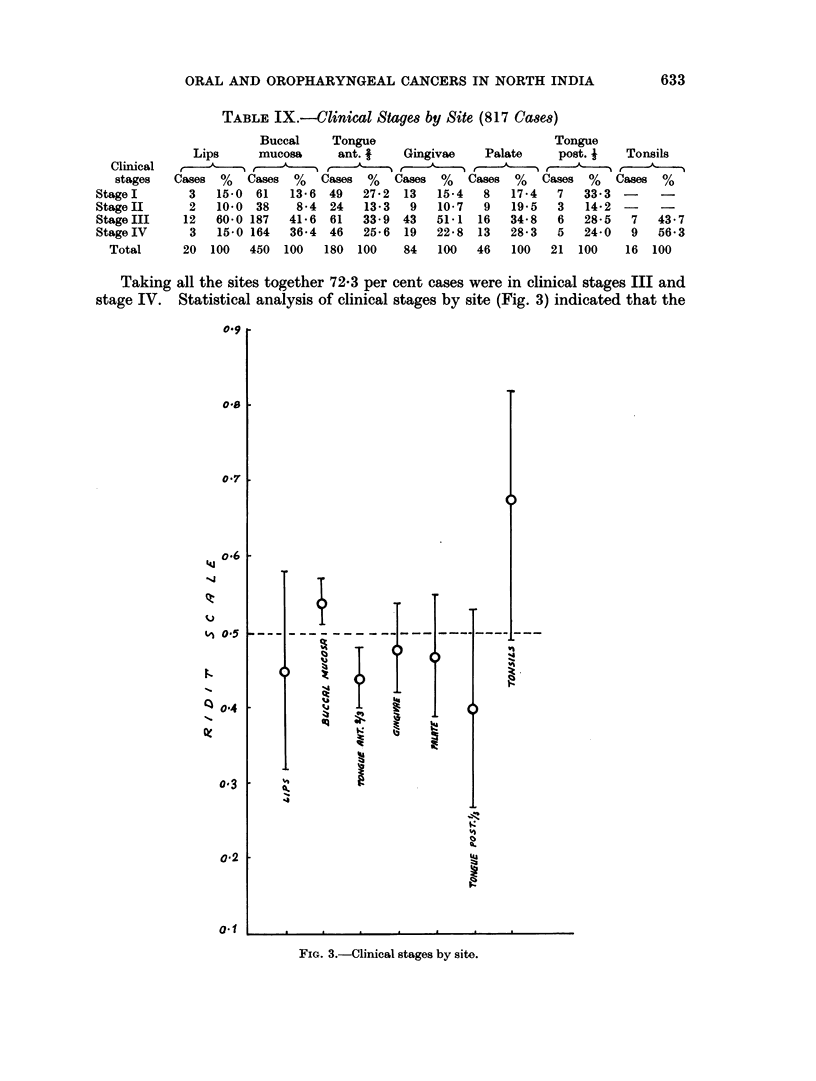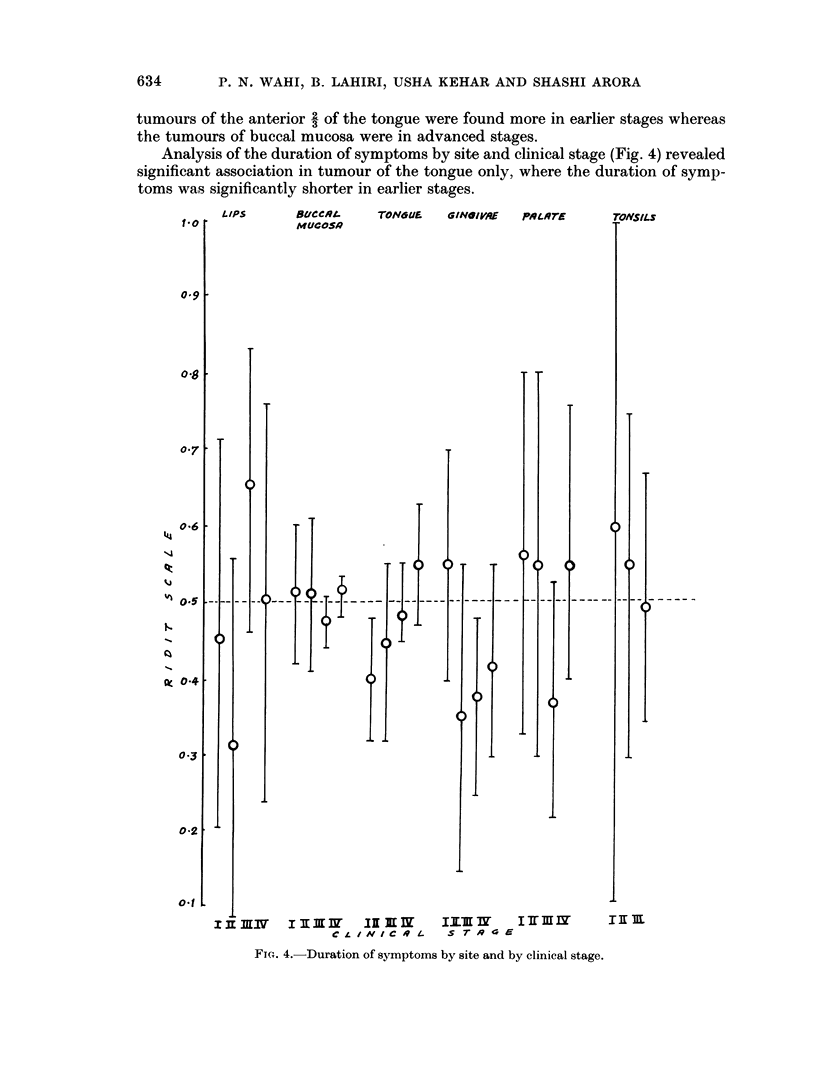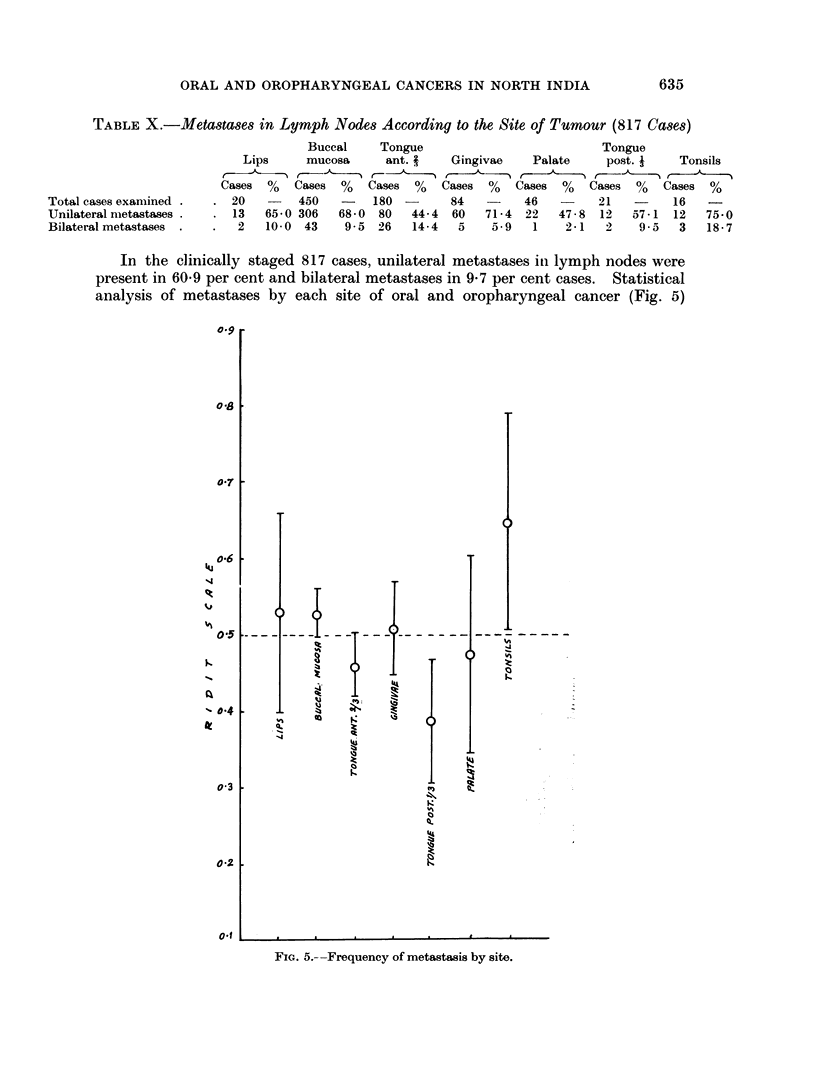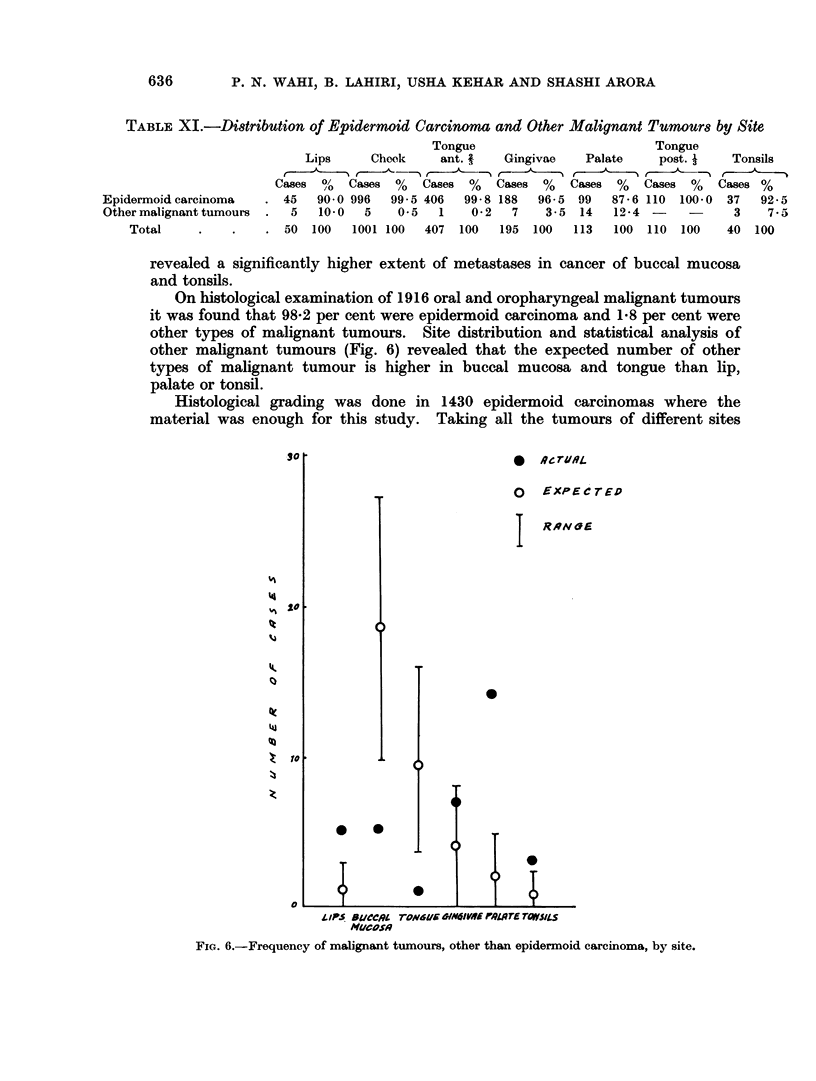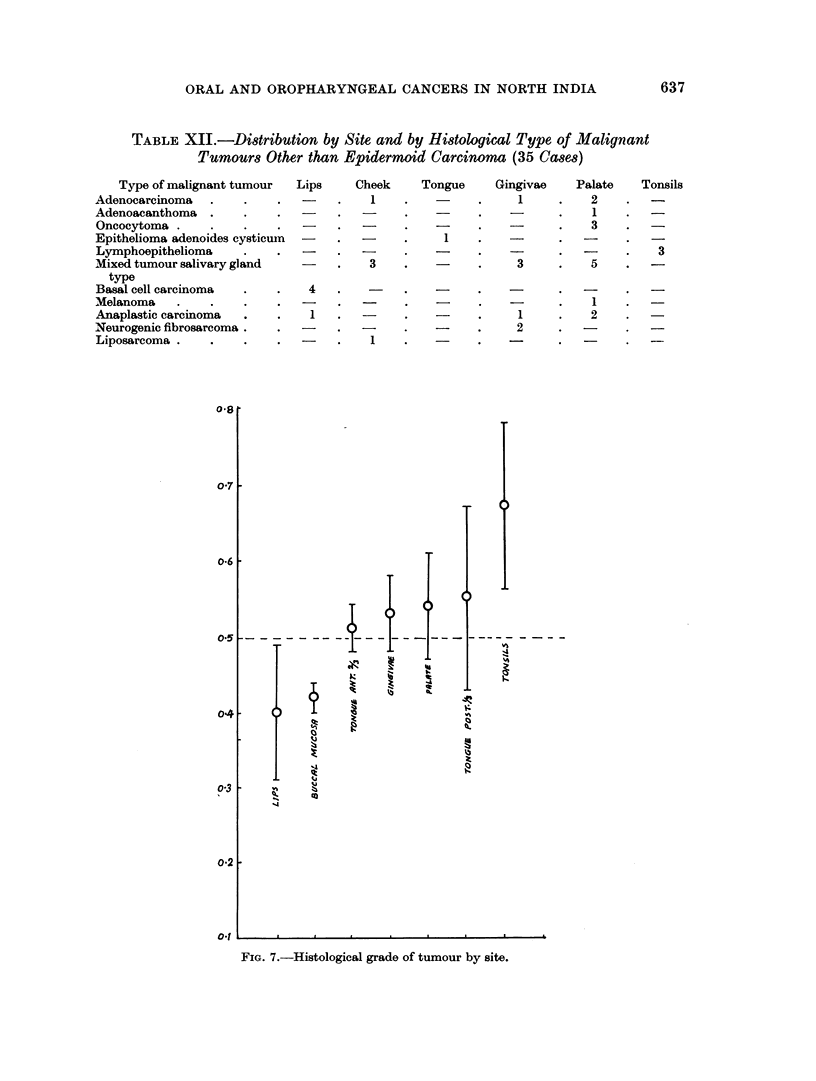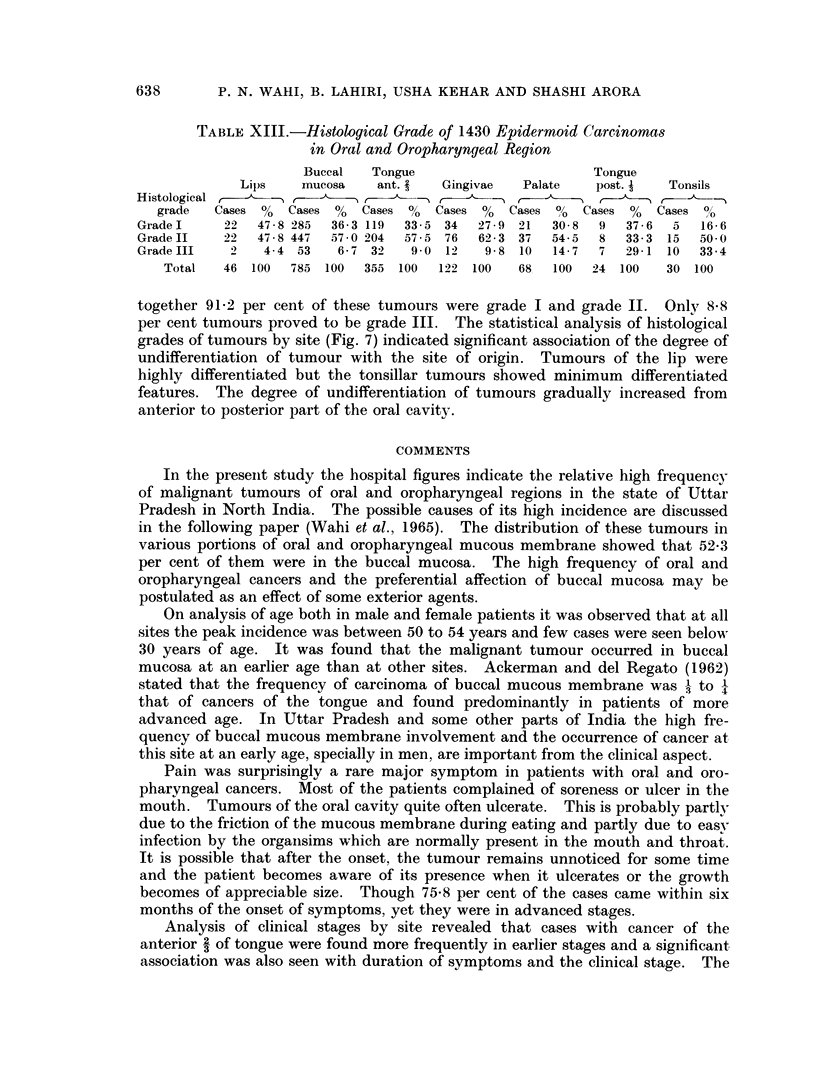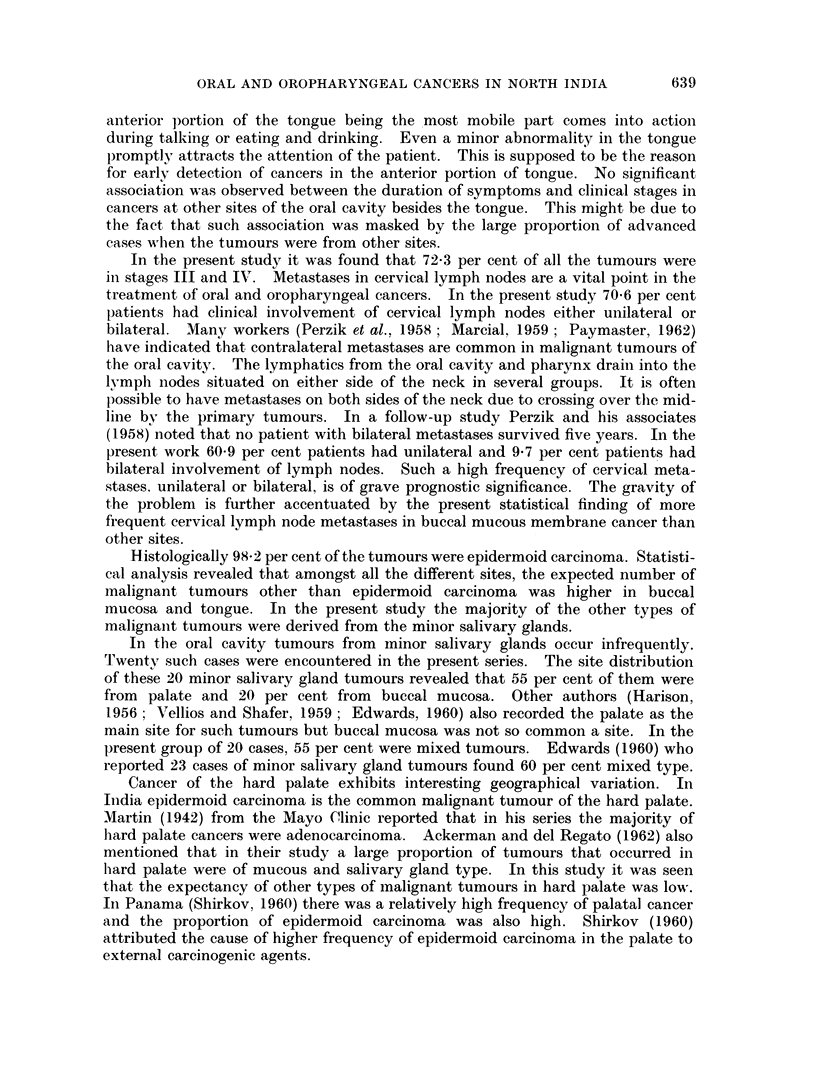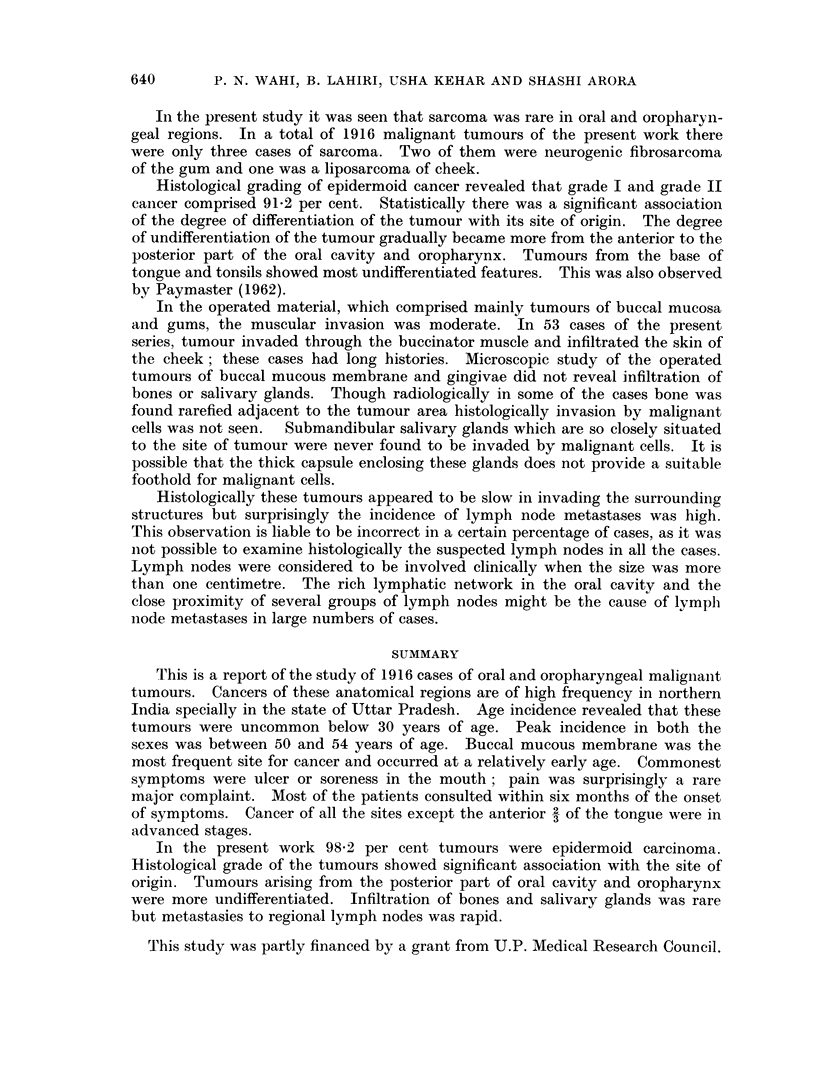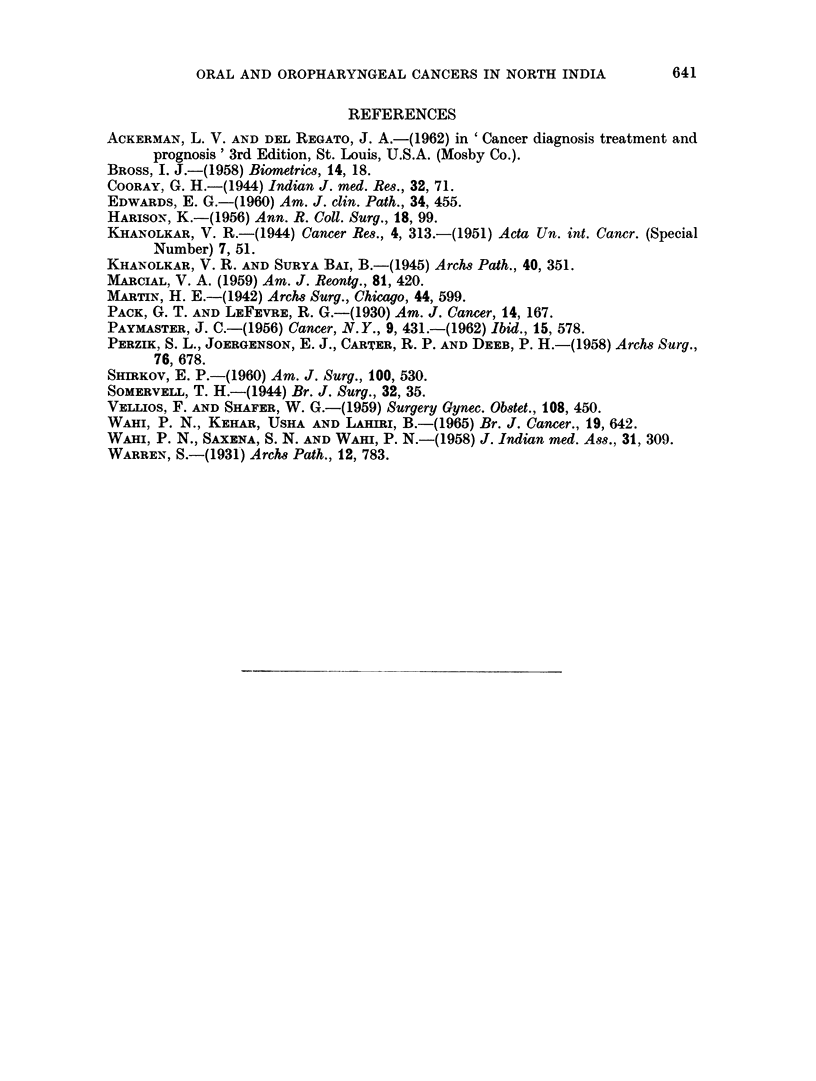# Oral and oropharyngeal cancers in North India.

**DOI:** 10.1038/bjc.1965.79

**Published:** 1965-12

**Authors:** P. N. Wahi, B. Lahiri, U. Kehar, S. Arora


### BRITISH JOURNAL OF CANCER

VOL. XIX DECEMBER, 1965 NO. 4

### ORAL AND OROPHARYNGEAL CANCERS IN NORTH INDIA

P. N. WAHI, B. LAHIRI, USHA KEHAR AND SHASHI ARORA

*From the Department of Pathology, Sarojini Naidu Medical College, Agra, India*

Received for publication June 21, 1965

TUMOURS of oral and oropharyngeal regions are the commonest malignant tumours in North India. A number of studies (Khanolkar, 1944, 1951 ; Paymaster, 1956 ; Wahi *et al.*, 1958) have brought out the close relationship of these tumours to environmental factors. The nature and the mode of use of the offending agents which are thought to be responsible for cancer in these regions vary from place to place. Likewise the anatomical location of tumours and their clinicopathological features are apt to be different. The purpose of this paper is to present the salient clinical and pathological features of these tumours as seen in the northern part of India, specially in the state of Uttar Pradesh.

### MATERIAL AND METHODS

During the years covered by this study (1950–62) 1916 cases of oral and oropharyngeal cancers were studied. Clinical and pathological features of these cases were noted and the tumours were clinically graded in 817 cases. The clinical staging was based on the following criteria :

Stage I. Where the main growth measured less than 2 cm. in diameter and metastases were absent.

Stage II. The lesion was 2 to 4 cm. in diameter and metastases were still absent.

Stage III. The lesion measured less than 4 cm. in diameter with unilateral metastases in the neck. The lymph nodes were mobile.

Stage IV. The lesion measured more than 4 cm. in diameter with or without metastases or with unilateral hard fixed lymph nodes in the neck or with bilateral metastases in the neck.

The histological grading of epidermoid carcinoma was done according to Shield Warren's system (Warren, 1931) based on the amount of keratinization, presence or absence of intercellular bridges and the number of mitotic figures in each high power field. Statistical study was done by using ridit analysis developed by Bross (1958).

Ridits are based on the observed distribution of response variable for a specified set of individuals. Ridits present a new application of an old idea (the probability transformation) and are closely related to distribution-free method based on ranks. Ridits indicate the degree of a particular class by number. The average ridit has probability interpretation—it is an estimate of the chance that an individual in a given class is "worse off" or "better off" than an individual in the reference class. The confidence interval on the average ridit involves the probability of a probability statement being true.

### OBSERVATIONS

TABLE I.—*Frequency of Malignant Tumours at Different Sites (Total of 6010 Cases)*

| Site | Number of cases | Percentage |
|---|---|---|
| Female genital system | 2159 | 35·9 |
| Oral cavity and oropharynx | 1916 | 31·9 |
| Male genital system | 327 | 5·4 |
| Skin | 264 | 4·4 |
| Breast | 250 | 4·2 |
| Gastrointestinal tract | 224 | 3·7 |
| Miscellaneous | 870 | 14·5 |
| Total | 6010 | 100 |

The analysis of the malignant tumours studied in the Department of Pathology (Table I) revealed the relative high frequency (31·9 per cent) of cancers of oral and oropharyngeal regions. It was a little less than the malignant tumours of female genital system (35·9 per cent). Tumours of gastrointestinal tract was as low as 3·7 per cent.

Table II gives the analysis of oral and oropharyngeal cancers by site. It is seen that buccal mucosa is the commonest site of involvement (52·3 per cent).

Table III indicates a regional preponderance of these tumours for different sites.

TABLE II.—*Distribution of Oral and Oropharyngeal Cancers by Site*

| Site | Number of cases | Percentage |
|---|---|---|
| Buccal mucosa | 1001 | 52·3 |
| Tongue-anterior ⅔ | 407 | 21·2 |
| Tongue-posterior ⅓ | 110 | 5·7 |
| Gingivae | 195 | 10·2 |
| Palate—hard | 105 | 5·5 |
| Palate—soft | 8 | 0·4 |
| Lips | 50 | 2·6 |
| Tonsils | 40 | 2·1 |
| Total | 1916 | 100 |

TABLE III.—*Frequency of Oral Cancer by Site as Reported by Different Observers*

| Place and authors | Total cases | Lips (%) | Buccal mucosa (%) | Tongue (%) | Gingivae (%) | Palate (%) |
|---|---|---|---|---|---|---|
| Bombay (India), Khanolkar (1944) | 1000 | 1·7 | 16·5 | 52·2 | 6·0 | 6·2 |
| Ceylon, Cooray (1944) | 274 | 13·2 | 48·5 | 15·7 | 15·7 | 5·4 |
| Visakhapatnam (India), Khanolkar and Surya Bai (1945) | 285 | 7·0 | 15·4 | 27·7 | 4·9 | 36·8 |
| Travancore (India), Somervell (1944) | 4497 | 6·0 | 45·5 | 13·0 | 35·0 | — |
| New York Memorial Hospital, Pack and LeFevre (1930) | 2862 | 20·7 | 12·7 | 28·2 | 7·0 | 10·7 |
| Agra (India), present report | 1916 | 2·6 | 52·3 | 26·9 | 10·2 | 5·9 |

The Bombay population has a high incidence of tongue cancer while in Ceylon, Travancore and Agra buccal mucous membrane and gingivae are the common sites of involvement. Figures of Memorial Hospital New York, reveal lips and tongue as the common sites.

Age break up of male and female patients (Table IV and V) according to site showed a low incidence of these tumours in the age group under 30 years. The incidence rate showed an upward trend from 30 years onwards and the peak was between 50 to 54 years of age. Gradual decline follows after that. Under 20 years of age there were 3 cases of carcinoma of buccal mucosa and 2 cases of palatal cancer. All the 5 cases were male. The youngest patient in the present

TABLE IV.—*Age Break Up of Male Patients of Oral and Oropharyngeal Cancer by Site (Total* 1351 *Cases)*

| Age in years | Lips | | Buccal mucosa | | Tongue ant. ⅔ | | Gingivae | | Palate | | Tongue post. ⅓ | | Tonsils | |
|---|---|---|---|---|---|---|---|---|---|---|---|---|---|---|
| | Cases | % | Cases | % | Cases | % | Cases | % | Cases | % | Cases | % | Cases | % |
| Below 20 | — | — | 3 | 0·5 | — | — | — | — | 2 | 2·2 | — | — | — | — |
| 21–24 | 1 | 2·8 | 7 | 1·0 | 2 | 0·6 | 1 | 0·8 | 1 | 1·1 | — | — | — | — |
| 25–29 | 1 | 2·8 | 22 | 3·3 | 7 | 2·3 | 5 | 4·3 | 4 | 4·4 | — | — | — | — |
| 30–34 | 2 | 5·7 | 68 | 10·1 | 29 | 9·3 | 8 | 6·8 | 4 | 4·4 | — | — | 2 | 5·9 |
| 35–39 | 4 | 11·4 | 73 | 10·9 | 35 | 11·2 | 9 | 7·7 | 4 | 4·4 | — | — | 4 | 11·7 |
| 40–44 | 2 | 5·7 | 125 | 18·8 | 50 | 16·1 | 16 | 13·7 | 12 | 13·2 | 22 | 23·4 | 7 | 20·6 |
| 45–49 | 1 | 2·8 | 85 | 12·8 | 43 | 13·9 | 16 | 13·7 | 9 | 9·9 | 15 | 16·0 | 5 | 14·7 |
| 50–54 | 12 | 34·3 | 125 | 18·8 | 56 | 18·0 | 24 | 20·6 | 20 | 22·0 | 32 | 34·0 | 10 | 29·5 |
| 55–59 | 5 | 14·4 | 55 | 8·2 | 32 | 10·3 | 16 | 13·7 | 11 | 12·1 | 15 | 16·0 | — | — |
| 60–64 | 5 | 14·4 | 76 | 11·3 | 37 | 11·9 | 11 | 9·4 | 14 | 15·3 | 6 | 6·4 | 5 | 14·7 |
| 65–69 | 2 | 5·7 | 17 | 2·5 | 9 | 2·9 | 4 | 3·4 | 5 | 5·5 | 3 | 3·2 | 1 | 2·9 |
| 70–74 | — | — | 9 | 1·3 | 9 | 2·9 | 7 | 5·9 | 4 | 4·4 | 1 | 1·0 | — | — |
| 75–79 | — | — | 3 | 0·4 | 1 | 0·3 | — | — | 1 | 1·1 | — | — | — | — |
| 80 and above | — | — | 1 | 0·1 | 1 | 0·3 | — | — | — | — | — | — | — | — |
| Total | 35 | 100 | 669 | 100 | 311 | 100 | 117 | 100 | 91 | 100 | 94 | 100 | 34 | 100 |

TABLE V.—*Age Break Up of Female Patients of Oral and Oropharyngeal Cancers by Site (Total* 565 *Cases)*

| Age in years | Lips | | Buccal mucosa | | Tongue ant. ⅔ | | Gingivae | | Palate | | Tongue post. ⅓ | | Tonsils | |
|---|---|---|---|---|---|---|---|---|---|---|---|---|---|---|
| | Cases | % | Cases | % | Cases | % | Cases | % | Cases | % | Cases | % | Cases | % |
| Below 20 | — | — | — | — | — | — | — | — | — | — | — | — | — | — |
| 21–24 | — | — | 4 | 1·2 | 1 | 1·0 | 2 | 2·6 | 4 | 18·2 | — | — | — | — |
| 25–29 | — | — | 5 | 1·5 | 4 | 4·2 | 1 | 1·3 | — | — | — | — | 1 | 16·7 |
| 30–34 | 2 | 13·3 | 23 | 6·9 | 7 | 7·3 | 8 | 10·3 | 1 | 4·6 | — | — | 1 | 16·7 |
| 35–39 | 2 | 13·3 | 30 | 9·0 | 10 | 10·4 | 3 | 3·8 | 3 | 13·6 | — | — | 1 | 16·7 |
| 40–44 | 5 | 33·3 | 66 | 20·0 | 10 | 10·4 | 7 | 9·0 | 7 | 31·8 | 1 | 6·2 | 1 | 16·7 |
| 45–49 | — | — | 50 | 15·1 | 18 | 18·8 | 9 | 11·5 | 3 | 13·6 | 2 | 12·5 | 1 | 16·7 |
| 50–54 | 3 | 20·0 | 71 | 21·4 | 21 | 21·8 | 23 | 29·5 | 3 | 13·6 | 7 | 43·9 | — | — |
| 55–59 | — | — | 32 | 9·6 | 7 | 7·3 | 5 | 6·4 | 1 | 4·6 | 1 | 6·2 | 1 | 16·7 |
| 60–64 | 1 | 6·7 | 38 | 11·4 | 9 | 9·4 | 12 | 15·4 | — | — | 3 | 18·7 | — | — |
| 65–69 | 1 | 6·7 | 3 | 0·9 | 2 | 2·1 | 3 | 3·8 | — | — | — | — | — | — |
| 70–74 | — | — | 7 | 2·1 | 3 | 3·1 | 5 | 6·4 | — | — | 2 | 12·5 | — | — |
| 75–79 | — | — | 2 | 0·6 | 2 | 2·1 | — | — | — | — | — | — | — | — |
| 80 and above | 1 | 6·7 | 1 | 0·3 | 2 | 2·1 | — | — | — | — | — | — | — | — |
| Total | 15 | 100 | 332 | 100 | 96 | 100 | 78 | 100 | 22 | 100 | 16 | 100 | 6 | 100 |

series was 14 years of age. There were 6 cases who were aged above 79 years, of which 2 were male and 4 female patients. In these cases lesions were in the buccal mucous membrane, lip or in the anterior portion of the tongue. In the present study the eldest patient was 87 years old.

Statistical analysis of the age incidence of both the sexes separately (Fig. 1) showed that in males the cancer of buccal mucosa tended to occur at earlier age while the malignant tumours of the posterior $\frac{1}{3}$ of the tongue and palate occurred

FIG. 1.—Age distribution by site and sex.

in advanced age. In females it was found that cancer of the posterior $\frac{1}{3}$ of the tongue had the tendency to arise in late years of life.

TABLE VI.—*Duration of Symptoms when the Patient First Visited Hospital* (1916 *Cases*)

| Duration in months | Lips | | Buccal mucosa | | Tongue | | Gingivae | | Palate | | Tonsils | |
|---|---|---|---|---|---|---|---|---|---|---|---|---|
| | Cases | % | Cases | % | Cases | % | Cases | % | Cases | % | Cases | % |
| 1–3 | 23 | 46·0 | 436 | 43·6 | 240 | 46·5 | 99 | 50·8 | 48 | 42·5 | 16 | 40·0 |
| 4–6 | 14 | 28·0 | 312 | 31·1 | 167 | 32·4 | 55 | 28·3 | 30 | 26·6 | 14 | 35·0 |
| 7–9 | 8 | 16·0 | 81 | 8·1 | 34 | 6·6 | 14 | 7·2 | 9 | 8·0 | 4 | 10·0 |
| 10–12 | 3 | 6·0 | 88 | 8·8 | 36 | 7·0 | 11 | 5·7 | 10 | 8·9 | 5 | 12·5 |
| 13–15 | — | — | 5 | 0·5 | 2 | 0·3 | 3 | 1·5 | — | — | — | — |
| 16–18 | — | — | 28 | 2·8 | 12 | 2·3 | 4 | 2·0 | 2 | 1·7 | 1 | 2·5 |
| 19–21 | — | — | 3 | 0·3 | 1 | 0·1 | 3 | 1·5 | 2 | 1·7 | — | — |
| 22–24 | 1 | 2·0 | 21 | 2·1 | 15 | 2·9 | 4 | 2·0 | 6 | 5·3 | — | — |
| above 2 years | 1 | 2·0 | 27 | 2·7 | 10 | 1·9 | 2 | 1·0 | 6 | 5·3 | — | — |
| Total | 50 | 100 | 1001 | 100 | 517 | 100 | 195 | 100 | 113 | 100 | 40 | 100 |

The majority of the patients in this study came to the hospital within 6 months of the onset of symptoms. There were 46 patients with a prolonged history. Some felt abnormal feelings in their mouth for more than 2 years to 20 years. These abnormal feelings in the mouth were of various types, e.g. soreness or ulcer in the mouth (19 cases), growth (20 cases), history of the presence of a white area in the buccal mucous membrane (2 cases), and the remaining 5 had some vague symptoms.

On statistical analysis, the duration of symptoms according to the site (Fig. 2) showed no significant differences. The major symptom of the patient which was the first complaint and of maximum duration has been analysed in Table VII.

The common presenting symptoms were soreness and ulcer in the mouth. Growth was noticed by 56 per cent cases of lip cancer. A white patch was the complaint of 1·7 per cent cases of buccal mucous membrane cancer. Pain was not a frequent symptom to the patients of oral cancer. This was relatively more common when the tumour was in the posterior $\frac{1}{3}$ of the tongue or in tonsils. Swelling of the neck due to involvement of regional lymph nodes was the chief complaint in 20·0 per cent cases of tonsillar cancer.

TABLE VII.—*Presenting Symptoms of* 1916 *Cases of Oral and Oropharyngeal Cancers by Site*

| Symptoms | Lips | | Buccal mucosa | | Tongue ant. $\frac{2}{3}$ | | Gingivae | | Palate | | Tongue post. $\frac{1}{3}$ | | Tonsils | |
|---|---|---|---|---|---|---|---|---|---|---|---|---|---|---|
| | Cases | % | Cases | % | Cases | % | Cases | % | Cases | % | Cases | % | Cases | % |
| Soreness and ulcer | 22 | 44·0 | 578 | 57·7 | 253 | 62·2 | 108 | 55·4 | 59 | 52·1 | 64 | 58·2 | 10 | 25·0 |
| Growth or swelling | 28 | 56·0 | 384 | 38·4 | 140 | 34·4 | 77 | 39·5 | 52 | 46·2 | 34 | 31·0 | 15 | 37·5 |
| White patch | — | — | 17 | 1·7 | — | — | — | — | — | — | — | — | — | — |
| Pain | — | — | 19 | 1·9 | 7 | 1·7 | 7 | 3·6 | 2 | 1·7 | 9 | 8·1 | 6 | 15·0 |
| Inability to open the mouth | — | — | 2 | 0·2 | — | — | — | — | — | — | — | — | — | — |
| Difficulty in swallowing and speaking | — | — | 1 | 0·1 | 2 | 0·5 | 1 | 0·5 | — | — | 3 | 2·7 | — | — |
| Swelling in the neck | — | — | — | — | 3 | 0·7 | 2 | 1·0 | — | — | — | — | 8 | 20·0 |
| Hoarseness of voice | — | — | — | — | 2 | 0·5 | — | — | — | — | — | — | 1 | 2·5 |
| Total | 50 | 100 | 1001 | 100 | 407 | 100 | 195 | 100 | 113 | 100 | 110 | 100 | 40 | 100 |

FIG. 2.—Duration of symptoms by site.

When the patients were first examined most of the oral and oropharyngeal tumours of different sites were either ulcerative or diffuse infiltrative type. The exophytic type of growth was seen in a few patients. Tumours which appeared as a small nodule were seen in buccal mucosa, tongue and gingivae. Leukoplakic appearance was noticed in two cases of carcinoma of buccal mucosa and one case of lip cancer. Table VIII gives the gross appearance of the tumour at various sites when first seen.

Clinical stage was accurately determined in 817 cases. Table IX shows clinical stages by site.

TABLE VIII.—*Gross Appearance of the Tumour when the Patients were First Examined (1327 Cases)*

| Gross appearance | Lips | | Buccal mucosa | | Tongue ant. $\frac{2}{3}$ | | Gingivae | | Palate | | Tongue post. $\frac{1}{3}$ | | Tonsils | |
|---|---|---|---|---|---|---|---|---|---|---|---|---|---|---|
| | Cases | % | Cases | % | Cases | % | Cases | % | Cases | % | Cases | % | Cases | % |
| Ulcerative | 17 | 44·8 | 390 | 54·7 | 182 | 60·0 | 74 | 55·2 | 35 | 48·7 | 16 | 61·6 | 18 | 42·9 |
| Diffuse infiltrative | 19 | 50·0 | 268 | 37·6 | 100 | 33·0 | 54 | 40·4 | 32 | 44·4 | 7 | 27 ·0 | 23 | 54·7 |
| Exophytic | 1 | 2·6 | 33 | 4·7 | 4 | 1·4 | 5 | 3·7 | 5 | 6·9 | 1 | 3·8 | 1 | 2·4 |
| Nodular | — | — | 19 | 2·7 | 17 | 5·6 | 1 | 0·7 | — | — | 2 | 7·6 | — | — |
| Leukoplakic | 1 | 2·6 | 2 | 0·3 | — | — | — | — | — | — | — | — | — | — |
| Total | 38 | 100 | 712 | 100 | 303 | 100 | 134 | 100 | 72 | 100 | 26 | 100 | 42 | 100 |

TABLE IX.—*Clinical Stages by Site* (817 *Cases*)

| Clinical stages | Lips | | Buccal mucosa | | Tongue ant. ⅔ | | Gingivae | | Palate | | Tongue post. ⅓ | | Tonsils | |
|---|---|---|---|---|---|---|---|---|---|---|---|---|---|---|
| | Cases | % | Cases | % | Cases | % | Cases | % | Cases | % | Cases | % | Cases | % |
| Stage I | 3 | 15·0 | 61 | 13·6 | 49 | 27·2 | 13 | 15·4 | 8 | 17·4 | 7 | 33·3 | — | — |
| Stage II | 2 | 10·0 | 38 | 8·4 | 24 | 13·3 | 9 | 10·7 | 9 | 19·5 | 3 | 14·2 | — | — |
| Stage III | 12 | 60·0 | 187 | 41·6 | 61 | 33·9 | 43 | 51·1 | 16 | 34·8 | 6 | 28·5 | 7 | 43·7 |
| Stage IV | 3 | 15·0 | 164 | 36·4 | 46 | 25·6 | 19 | 22·8 | 13 | 28·3 | 5 | 24·0 | 9 | 56·3 |
| Total | 20 | 100 | 450 | 100 | 180 | 100 | 84 | 100 | 46 | 100 | 21 | 100 | 16 | 100 |

Taking all the sites together 72·3 per cent cases were in clinical stages III and stage IV. Statistical analysis of clinical stages by site (Fig. 3) indicated that the

FIG. 3.—Clinical stages by site.

tumours of the anterior $\frac{2}{3}$ of the tongue were found more in earlier stages whereas the tumours of buccal mucosa were in advanced stages.

Analysis of the duration of symptoms by site and clinical stage (Fig. 4) revealed significant association in tumour of the tongue only, where the duration of symptoms was significantly shorter in earlier stages.

FIG. 4.—Duration of symptoms by site and by clinical stage.

TABLE X.—*Metastases in Lymph Nodes According to the Site of Tumour* (817 *Cases*)

| | Lips | | Buccal mucosa | | Tongue ant. ⅔ | | Gingivae | | Palate | | Tongue post. ⅓ | | Tonsils | |
|---|---|---|---|---|---|---|---|---|---|---|---|---|---|---|
| | Cases | % | Cases | % | Cases | % | Cases | % | Cases | % | Cases | % | Cases | % |
| Total cases examined . . | 20 | — | 450 | — | 180 | — | 84 | — | 46 | — | 21 | — | 16 | — |
| Unilateral metastases . . | 13 | 65·0 | 306 | 68·0 | 80 | 44·4 | 60 | 71·4 | 22 | 47·8 | 12 | 57·1 | 12 | 75·0 |
| Bilateral metastases . . | 2 | 10·0 | 43 | 9·5 | 26 | 14·4 | 5 | 5·9 | 1 | 2·1 | 2 | 9·5 | 3 | 18·7 |

In the clinically staged 817 cases, unilateral metastases in lymph nodes were present in 60·9 per cent and bilateral metastases in 9·7 per cent cases. Statistical analysis of metastases by each site of oral and oropharyngeal cancer (Fig. 5)

FIG. 5.--Frequency of metastasis by site.

TABLE XI.—*Distribution of Epidermoid Carcinoma and Other Malignant Tumours by Site*

| | Lips | | Cheek | | Tongue ant. ⅔ | | Gingivae | | Palate | | Tongue post. ⅓ | | Tonsils | |
|---|---|---|---|---|---|---|---|---|---|---|---|---|---|---|
| | Cases | % | Cases | % | Cases | % | Cases | % | Cases | % | Cases | % | Cases | % |
| Epidermoid carcinoma . | 45 | 90·0 | 996 | 99·5 | 406 | 99·8 | 188 | 96·5 | 99 | 87·6 | 110 | 100·0 | 37 | 92·5 |
| Other malignant tumours . | 5 | 10·0 | 5 | 0·5 | 1 | 0·2 | 7 | 3·5 | 14 | 12·4 | — | — | 3 | 7·5 |
| Total . . . . | 50 | 100 | 1001 | 100 | 407 | 100 | 195 | 100 | 113 | 100 | 110 | 100 | 40 | 100 |

revealed a significantly higher extent of metastases in cancer of buccal mucosa and tonsils.

On histological examination of 1916 oral and oropharyngeal malignant tumours it was found that 98·2 per cent were epidermoid carcinoma and 1·8 per cent were other types of malignant tumours. Site distribution and statistical analysis of other malignant tumours (Fig. 6) revealed that the expected number of other types of malignant tumour is higher in buccal mucosa and tongue than lip, palate or tonsil.

Histological grading was done in 1430 epidermoid carcinomas where the material was enough for this study. Taking all the tumours of different sites

FIG. 6.—Frequency of malignant tumours, other than epidermoid carcinoma, by site.

TABLE XII.—*Distribution by Site and by Histological Type of Malignant Tumours Other than Epidermoid Carcinoma* (35 *Cases*)

| Type of malignant tumour | Lips | Cheek | Tongue | Gingivae | Palate | Tonsils |
|---|---|---|---|---|---|---|
| Adenocarcinoma | — | 1 | — | 1 | 2 | — |
| Adenoacanthoma | — | — | — | — | 1 | — |
| Oncocytoma | — | — | — | — | 3 | — |
| Epithelioma adenoides cysticum | — | — | 1 | — | — | — |
| Lymphoepithelioma | — | — | — | — | — | 3 |
| Mixed tumour salivary gland type | — | 3 | — | 3 | 5 | — |
| Basal cell carcinoma | 4 | — | — | — | — | — |
| Melanoma | — | — | — | — | 1 | — |
| Anaplastic carcinoma | 1 | — | — | 1 | 2 | — |
| Neurogenic fibrosarcoma | — | — | — | 2 | — | — |
| Liposarcoma | — | 1 | — | — | — | — |

FIG. 7.—Histological grade of tumour by site.

TABLE XIII.—*Histological Grade of* 1430 *Epidermoid Carcinomas in Oral and Oropharyngeal Region*

| Histological grade | Lips | | Buccal mucosa | | Tongue ant. ⅔ | | Gingivae | | Palate | | Tongue post. ⅓ | | Tonsils | |
|---|---|---|---|---|---|---|---|---|---|---|---|---|---|---|
| | Cases | % | Cases | % | Cases | % | Cases | % | Cases | % | Cases | % | Cases | % |
| Grade I | 22 | 47·8 | 285 | 36·3 | 119 | 33·5 | 34 | 27·9 | 21 | 30·8 | 9 | 37·6 | 5 | 16·6 |
| Grade II | 22 | 47·8 | 447 | 57·0 | 204 | 57·5 | 76 | 62·3 | 37 | 54·5 | 8 | 33·3 | 15 | 50·0 |
| Grade III | 2 | 4·4 | 53 | 6·7 | 32 | 9·0 | 12 | 9·8 | 10 | 14·7 | 7 | 29·1 | 10 | 33·4 |
| Total | 46 | 100 | 785 | 100 | 355 | 100 | 122 | 100 | 68 | 100 | 24 | 100 | 30 | 100 |

together 91·2 per cent of these tumours were grade I and grade II. Only 8·8 per cent tumours proved to be grade III. The statistical analysis of histological grades of tumours by site (Fig. 7) indicated significant association of the degree of undifferentiation of tumour with the site of origin. Tumours of the lip were highly differentiated but the tonsillar tumours showed minimum differentiated features. The degree of undifferentiation of tumours gradually increased from anterior to posterior part of the oral cavity.

### COMMENTS

In the present study the hospital figures indicate the relative high frequency of malignant tumours of oral and oropharyngeal regions in the state of Uttar Pradesh in North India. The possible causes of its high incidence are discussed in the following paper (Wahi *et al.*, 1965). The distribution of these tumours in various portions of oral and oropharyngeal mucous membrane showed that 52·3 per cent of them were in the buccal mucosa. The high frequency of oral and oropharyngeal cancers and the preferential affection of buccal mucosa may be postulated as an effect of some exterior agents.

On analysis of age both in male and female patients it was observed that at all sites the peak incidence was between 50 to 54 years and few cases were seen below 30 years of age. It was found that the malignant tumour occurred in buccal mucosa at an earlier age than at other sites. Ackerman and del Regato (1962) stated that the frequency of carcinoma of buccal mucous membrane was ⅓ to ¼ that of cancers of the tongue and found predominantly in patients of more advanced age. In Uttar Pradesh and some other parts of India the high frequency of buccal mucous membrane involvement and the occurrence of cancer at this site at an early age, specially in men, are important from the clinical aspect.

Pain was surprisingly a rare major symptom in patients with oral and oropharyngeal cancers. Most of the patients complained of soreness or ulcer in the mouth. Tumours of the oral cavity quite often ulcerate. This is probably partly due to the friction of the mucous membrane during eating and partly due to easy infection by the organsims which are normally present in the mouth and throat. It is possible that after the onset, the tumour remains unnoticed for some time and the patient becomes aware of its presence when it ulcerates or the growth becomes of appreciable size. Though 75·8 per cent of the cases came within six months of the onset of symptoms, yet they were in advanced stages.

Analysis of clinical stages by site revealed that cases with cancer of the anterior ⅔ of tongue were found more frequently in earlier stages and a significant association was also seen with duration of symptoms and the clinical stage. The anterior portion of the tongue being the most mobile part comes into action during talking or eating and drinking. Even a minor abnormality in the tongue promptly attracts the attention of the patient. This is supposed to be the reason for early detection of cancers in the anterior portion of tongue. No significant association was observed between the duration of symptoms and clinical stages in cancers at other sites of the oral cavity besides the tongue. This might be due to the fact that such association was masked by the large proportion of advanced cases when the tumours were from other sites.

In the present study it was found that 72·3 per cent of all the tumours were in stages III and IV. Metastases in cervical lymph nodes are a vital point in the treatment of oral and oropharyngeal cancers. In the present study 70·6 per cent patients had clinical involvement of cervical lymph nodes either unilateral or bilateral. Many workers (Perzik *et al.*, 1958 ; Marcial, 1959 ; Paymaster, 1962) have indicated that contralateral metastases are common in malignant tumours of the oral cavity. The lymphatics from the oral cavity and pharynx drain into the lymph nodes situated on either side of the neck in several groups. It is often possible to have metastases on both sides of the neck due to crossing over the mid-line by the primary tumours. In a follow-up study Perzik and his associates (1958) noted that no patient with bilateral metastases survived five years. In the present work 60·9 per cent patients had unilateral and 9·7 per cent patients had bilateral involvement of lymph nodes. Such a high frequency of cervical metastases, unilateral or bilateral, is of grave prognostic significance. The gravity of the problem is further accentuated by the present statistical finding of more frequent cervical lymph node metastases in buccal mucous membrane cancer than other sites.

Histologically 98·2 per cent of the tumours were epidermoid carcinoma. Statistical analysis revealed that amongst all the different sites, the expected number of malignant tumours other than epidermoid carcinoma was higher in buccal mucosa and tongue. In the present study the majority of the other types of malignant tumours were derived from the minor salivary glands.

In the oral cavity tumours from minor salivary glands occur infrequently. Twenty such cases were encountered in the present series. The site distribution of these 20 minor salivary gland tumours revealed that 55 per cent of them were from palate and 20 per cent from buccal mucosa. Other authors (Harison, 1956 ; Vellios and Shafer, 1959 ; Edwards, 1960) also recorded the palate as the main site for such tumours but buccal mucosa was not so common a site. In the present group of 20 cases, 55 per cent were mixed tumours. Edwards (1960) who reported 23 cases of minor salivary gland tumours found 60 per cent mixed type.

Cancer of the hard palate exhibits interesting geographical variation. In India epidermoid carcinoma is the common malignant tumour of the hard palate. Martin (1942) from the Mayo Clinic reported that in his series the majority of hard palate cancers were adenocarcinoma. Ackerman and del Regato (1962) also mentioned that in their study a large proportion of tumours that occurred in hard palate were of mucous and salivary gland type. In this study it was seen that the expectancy of other types of malignant tumours in hard palate was low. In Panama (Shirkov, 1960) there was a relatively high frequency of palatal cancer and the proportion of epidermoid carcinoma was also high. Shirkov (1960) attributed the cause of higher frequency of epidermoid carcinoma in the palate to external carcinogenic agents.

In the present study it was seen that sarcoma was rare in oral and oropharyngeal regions. In a total of 1916 malignant tumours of the present work there were only three cases of sarcoma. Two of them were neurogenic fibrosarcoma of the gum and one was a liposarcoma of cheek.

Histological grading of epidermoid cancer revealed that grade I and grade II cancer comprised 91·2 per cent. Statistically there was a significant association of the degree of differentiation of the tumour with its site of origin. The degree of undifferentiation of the tumour gradually became more from the anterior to the posterior part of the oral cavity and oropharynx. Tumours from the base of tongue and tonsils showed most undifferentiated features. This was also observed by Paymaster (1962).

In the operated material, which comprised mainly tumours of buccal mucosa and gums, the muscular invasion was moderate. In 53 cases of the present series, tumour invaded through the buccinator muscle and infiltrated the skin of the cheek ; these cases had long histories. Microscopic study of the operated tumours of buccal mucous membrane and gingivae did not reveal infiltration of bones or salivary glands. Though radiologically in some of the cases bone was found rarefied adjacent to the tumour area histologically invasion by malignant cells was not seen. Submandibular salivary glands which are so closely situated to the site of tumour were never found to be invaded by malignant cells. It is possible that the thick capsule enclosing these glands does not provide a suitable foothold for malignant cells.

Histologically these tumours appeared to be slow in invading the surrounding structures but surprisingly the incidence of lymph node metastases was high. This observation is liable to be incorrect in a certain percentage of cases, as it was not possible to examine histologically the suspected lymph nodes in all the cases. Lymph nodes were considered to be involved clinically when the size was more than one centimetre. The rich lymphatic network in the oral cavity and the close proximity of several groups of lymph nodes might be the cause of lymph node metastases in large numbers of cases.

### SUMMARY

This is a report of the study of 1916 cases of oral and oropharyngeal malignant tumours. Cancers of these anatomical regions are of high frequency in northern India specially in the state of Uttar Pradesh. Age incidence revealed that these tumours were uncommon below 30 years of age. Peak incidence in both the sexes was between 50 and 54 years of age. Buccal mucous membrane was the most frequent site for cancer and occurred at a relatively early age. Commonest symptoms were ulcer or soreness in the mouth ; pain was surprisingly a rare major complaint. Most of the patients consulted within six months of the onset of symptoms. Cancer of all the sites except the anterior $\frac{2}{3}$ of the tongue were in advanced stages.

In the present work 98·2 per cent tumours were epidermoid carcinoma. Histological grade of the tumours showed significant association with the site of origin. Tumours arising from the posterior part of oral cavity and oropharynx were more undifferentiated. Infiltration of bones and salivary glands was rare but metastasies to regional lymph nodes was rapid.

This study was partly financed by a grant from U.P. Medical Research Council.

### REFERENCES

ACKERMAN, L. V. AND DEL REGATO, J. A.—(1962) in 'Cancer diagnosis treatment and prognosis' 3rd Edition, St. Louis, U.S.A. (Mosby Co.).
BROSS, I. J.—(1958) *Biometrics*, **14**, 18.
COORAY, G. H.—(1944) *Indian J. med. Res.*, **32**, 71.
EDWARDS, E. G.—(1960) *Am. J. clin. Path.*, **34**, 455.
HARISON, K.—(1956) *Ann. R. Coll. Surg.*, **18**, 99.
KHANOLKAR, V. R.—(1944) *Cancer Res.*, **4**, 313.—(1951) *Acta Un. int. Cancr.* (Special Number) **7**, 51.
KHANOLKAR, V. R. AND SURYA BAI, B.—(1945) *Archs Path.*, **40**, 351.
MARCIAL, V. A. (1959) *Am. J. Reontg.*, **81**, 420.
MARTIN, H. E.—(1942) *Archs Surg., Chicago*, **44**, 599.
PACK, G. T. AND LEFEVRE, R. G.—(1930) *Am. J. Cancer*, **14**, 167.
PAYMASTER, J. C.—(1956) *Cancer, N.Y.*, **9**, 431.—(1962) *Ibid.*, **15**, 578.
PERZIK, S. L., JOERGENSON, E. J., CARTER, R. P. AND DEEB, P. H.—(1958) *Archs Surg.*, **76**, 678.
SHIRKOV, E. P.—(1960) *Am. J. Surg.*, **100**, 530.
SOMERVELL, T. H.—(1944) *Br. J. Surg.*, **32**, 35.
VELLIOS, F. AND SHAFER, W. G.—(1959) *Surgery Gynec. Obstet.*, **108**, 450.
WAHI, P. N., KEHAR, USHA AND LAHIRI, B.—(1965) *Br. J. Cancer.*, **19**, 642.
WAHI, P. N., SAXENA, S. N. AND WAHI, P. N.—(1958) *J. Indian med. Ass.*, **31**, 309.
WARREN, S.—(1931) *Archs Path.*, **12**, 783.